# Complete genome sequence of *Bifidobacterium animalis* subsp. *lactis* BI040, a probiotic strain with multiple health benefits from the NORDBIOTIC collection

**DOI:** 10.1128/mra.01117-24

**Published:** 2025-01-17

**Authors:** Agnieszka K. Szczepankowska, Bożena Cukrowska, Tamara Aleksandrzak-Piekarczyk

**Affiliations:** 1 Institute of Biochemistry and Biophysics, Polish Academy of Sciences, Warsaw, Poland; 2 The Children Memorial Health Institute, Department of Pathomorphology, Warsaw, Poland; University of Maryland School of Medicine, Baltimore, Maryland, USA

**Keywords:** *Bifidobacterium*, stool sample, vitamin B6 synthesis, gut health, probiotics, irritable bowel syndrome, respiratory viral infections, succinate, anti-obesity effects, bile salt hydrolase

## Abstract

The complete genome of *Bifidobacterium animalis* subsp. *lactis* BI040—a human stool isolate, was sequenced using Illumina and Oxford Nanopore technologies. The BI040 genome is composed of a circular 1,944,141-bp chromosome which carries genes potentially involved in vitamin synthesis and gut health.

## ANNOUNCEMENT

*Bifidobacterium animalis* strains are commonly recognized for their probiotic potential and ability to colonize the human intestine (1). *B. animalis* subsp. *lactis* BI040 from the NORDBIOTIC collection was originally isolated from a human stool sample and has been deposited in the DSMZ Collection (DSM33812). In clinical trials, BI040 demonstrated beneficial effects for patients with irritable bowel syndrome (IBS) (2) and respiratory viral infections (3).

The strain was provided from the NORDBIOTIC collection and cultured overnight from a single colony at 37°C in MRS medium (Oxoid) under anaerobic conditions. DNA was isolated using a cetyltrimethylammonium bromide/lysozyme method (4) with a lysozyme/mutanolysin pre-digestion step (5) and sequenced independently with Illumina and Oxford Nanopore technologies.

Illumina library was prepared with the NEB Ultra II FS kit (New England Biolabs) and sequenced in paired-end mode (2 × 300 bp) on the MiSeq platform (Illumina). Raw data were trimmed and filtered with FASTQC (v.0.12.0) (6) and fastp (v.0.23.2) (7), resulting in 666,502 final reads of average 311 × coverage and 296,897,927 nt.

Long-read sequencing library was created using sheared and size-selected (>10 kb) DNA fragments (Short Read Eliminator kit; Circulomics) and the SQK-LSK109 native barcoding expansion kit (EXP-NBD103), loaded on an R9.4.1 flowcell and sequenced on the GridION sequencer (Oxford Nanopore Technologies). Raw ONT reads were generated using Guppy (v.6.1.3) (ONT) in super accuracy mode and filtered with NanoFilt (v.2.8.0) (8) to eliminate short (<1 kb) and low-quality (QS <12) reads, and with Porechop (v.0.2.4) (https://github.com/rrwick/Porechop) to remove residual adapters. Sequence quality was checked using NanoPlot (v.1.41.6) (8). Final Nanopore sequencing resulted in 90,371 reads with an average length of 8,657 nt, *N*_50_ value of 12,410, and 198 × coverage. Default parameters were used for all software.

ONT long-reads were assembled using Trycycler (v.0.5.3) (9) and Flye (v.2.9) (10), Unicycler (v.0.4.8) (11), Raven (v.1.8.1) (12), and Miniasm (v.0.3-r179) (13), reconciled and circularized with Trycycler, and polished using Racon (v.1.5.0) (14) and Medaka (v.1.7.2) (15). To obtain a high-quality consensus sequence, the ONT assembly was corrected with Illumina data using Polypolish (v.0.5.0) (16) and POLCA (v.4.0.5) (17), resulting in a circular, 1,944,141 bp chromosome with 509.0 coverage and a GC content of 60.5%. Coding sequence (CDS) prediction and gene annotation were performed using the NCBI Prokaryotic Genome Annotation Pipeline (v.6.6) (18), identifying 1,543 predicted CDSs, 52 tRNAs, 12 rRNAs, and 3 ncRNAs. A complete set of genes for *de novo* vitamin B6 synthesis via deoxyxylulose 5-phosphate-independent pathway was identified, indicating potential nutritional benefits for BI040-fermented food products. Additionally, the presence of *sucCD* genes encoding succinyl-CoA synthase for the conversion of succinyl-CoA to succinic acid was observed. Succinate, a microbiome-produced by-product, plays a role in maintaining gut equilibrium and in inducing pro-inflammatory responses to manage local stress (19), potentially aiding in constipation relief, a condition inversely correlated with obesity-related metabolic disturbances. We identified gene encoding bile salt hydrolase [EC 3.5.1.24], which catalyzes the deconjugation of glycine- and taurine-linked bile salts, reducing their reabsorption efficiency compared to conjugated bile salts, and potentially contributing to lower serum cholesterol levels (20).

## Data Availability

Complete sequencing data has been deposited in GenBank under BioProject PRJNA1071652 and BioSample SAMN39639087. Whole-genome data are available under accession number CP147973. lllumina SRA reads are available under accession number SRX23536629. Oxford Nanopore SRA reads are available under accession number SRX23536628.
